# Rapid biodegradation of polycyclic aromatic hydrocarbons (PAHs) using effective *Cronobacter sakazakii* MM045 (KT933253)

**DOI:** 10.1016/j.mex.2017.02.003

**Published:** 2017-02-20

**Authors:** Zubairu Darma Umar, Nor Azwady Abd. Aziz, Syaizwan Zahmir Zulkifli, Muskhazli Mustafa

**Affiliations:** a Department of Biology, Faculty of Science, Universiti Putra Malaysia, 43400 Serdang, Selangor, Malaysia; b Department of Microbiology, Umaru Musa Yar’adua University Katsina, P.M.B. 2218, Katsina State, Nigeria

**Keywords:** Rapid PAHs biodegradation, Biodegradation, Phenanthrene, Pyrene, Optimization, *C. sakazakii* MM045

## Abstract

Polycyclic Aromatic Hydrocarbons (PAHs) are complex and widely distributed environmental pollutants that can affect living ecosystems. This study was conducted to rapidly degrade phenanthrene and pyrene representing low and high molecular weight of PAHs, respectively. *Cronobacter sakazakii* MM045 (KT933253) was identified from used engine oil of contaminated soil. PAHs biodegradation was carried out using 2,6-dichlorophenol indophenol (DCPIP) assay. Biodegradation influencing factors including agitation, temperature, pH, inoculums volume and salinity were enhanced using Response Surface Methodology (RSM) by Central Composite Design (CCD). Phenanthrene and pyrene biodegrading metabolites were identified using gas chromatography mass spectrophotometer (GCMS).

• Initial biodegradation indicated 75.2% and 54.3% phenanthrene and pyrene degraded by *C. sakazakii* MM045 within 24 h. After CCD optimisation, 100% degradation was achieved for each of the phenanthrene and pyrene, resulting in the formation of intermediate metabolites.
• The identified phenanthrene metabolites were 3,4-dihydroxyphenathrene, phthalic acid, pyruvic acid, acetic acid and oxalic acid. Pyrene intermediates comprised pyrene *cis*-4,5-dihydrodiol, 3,4-dihydroxyphenanthrene, phthalic acid, pyruvic acid, acetic acid and lactic acid.
• *Cronbacter sakazakii* MM045 was proven to be rapid and effective in degrading PAHs within 24 h despite the unavailability of existing literatures on PAHs biodegradation.

## Graphical abstract

**Figure:**
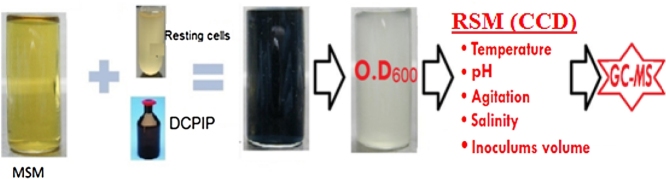

## Method details

### Chemicals and bacterium maintenance (Steps 1–3)

Phenanthrene and pyrene (96%–98% purity) were purchased from Sigma-Aldrich and Merck Chemicals, respectively. Other analytical grade chemicals and media were purchased from standard manufacturers and each PAH was dissolved in petroleum ether followed by filter (0.2 μm) sterilisation. Phosphate buffer saline (PBS) containing 0.24 g/L KH_2_PO_4_, 1.44 g/L Na_2_HPO_4_, 0.20 g/L KCl, 8.00 g/L NaCl, pH 7.4 was prepared using sterile deionised water. Mineral salts medium (MSM) (0.20 g/L ZnSO_4_•7H_2_O; 0.20 g/L MgSO_4_•7H_2_O, 2.00 g/L NH_4_Cl, 1.50 g/L KH_2_PO_4_, 9.00 g/L Na_2_HPO_4_, 10.00 μg/L CoSO_4_•7H_2_O, 3.00 g/L MnSO_4_•H_2_O, 5.00 g/L Ferric citrate, 0.01 g/L Titriplex III, pH 7.4) was prepared and employed in the biodegradation studies. Triplicate experiments were carried out for each treatment and un-inoculated media were used as negative controls.

### Organisms and growth conditions

Previously identified *C. sakazakii* MM045 by Darma et al. [12] from used engine oil of contaminated soil was used in this study. Initially, *Cronobacter* resting cells were prepared and maintained in PBS at 4 °C for biodegradation studies [36]. Concentrations for Phenanthrene (500 mg/L) and Pyrene (250 mg/L) were chosen from the previous data obtained in the standard calibration curves [12]. Prepared MSM was supplemented with 500 mg/L phenanthrene and 250 mg/L pyrene separately, which were then mixed with 200 mg/L DCPIP to be utilised as PAHs degradation indicators [9]. The initial degradation before the RSM was carried out using 5% (v/v) *C. sakazakii* resting cells and incubation conditions of 37 °C and 200 rpm for 24 h, which were determined using spectrophotometer [15].

### RSM (CCD) optimisation (Steps 4 & 5)

Biodegradation factors namely agitation, temperature, pH, inoculums volume (IV), and salinity were selected to influence the biodegradation of phenanthrene and pyrene using *C. sakazakii* MM045. The result of combined factors was enhanced based on CCD optimisation using design expert software, version 6.0 (Stat-Ease Inc., Minneapolis, USA). A total of 50 experimental treatments were carried out for each phenanthrene and pyrene (Table 1). Quadratic model significance was determined by *t*-test and multiple regressions [29]. The response from *Cronobacter sakazakii* MM045 degradation is the dependent variable, whereas agitation, temperature, pH, IV and salinity are the independent variables. Correlation among variables was assessed based on second-order polynomial and the quadratic model was expressed mathematically.

(1) *Y* *=* *β*_0_ + *β*_1_*x*_1_ + *β*_2_*x*_2_ + *β*_3_*x*_3_ + *β*_4_*x*_4_ _+_ *β*_5_*x*_5_ _+_ *β*_11_*x*_1_^2^ + *β*_22_*x*_2_^2^ + *β*_33_*x*_3_^2^ + *β*_44_*x*_4_^2^ +* β*_55_*x*_5_^2^ +* β*_12_*x*_1_*x*_2_ + *β*_13_*x*_1_*x*_3_ +* β*_14_*x*_1_*x*_4_ + *β*_15_*x*_1_*x*_5_ +* β*_23_*x*_2_*x*_3_ +* β*_24_*x*_2_*x*_4_ +* β*_25_*x*_2_*x*_5_ + *β*_34_*x*_3_*x*_4_ + *β*_35_*x*_2_*x*_5_ +* β*_45_*x*_4_*x*_5,_

Where *Y* =% PAH degradation; *β*_0_ = interception coefficient; *β*_1_, *β*_2_, *β*_3,_ *β*_4,_ *β*_5_ = linear coefficients; *β*_11_, *β*_22_, *β*_33_, *β*_44_, *β*_55_ = quadratic coefficients; *β*_12_, *β*_13_, *β*_14_, *β*_15_, *β*_23_, *β*_24,_ *β*_25_, *β*_34_, *β*_35,_ *β*_45_ = interactions coefficient; *x*_1_, *x*_2_, *x*_3,_ *x*_4_, *x*_5_ = rpm, temperature, pH, IV and salinity. Result was finally validated based on prediction by numerical optimization.

### Results of phenanthrene RSM optimisation

Quadratic model as interpreted in Eq. (1) was mathematically presented considering the 50 CCD experimental treatment of phenanthrene degradation by *C. sakazakii* MM045 (Table 1).

(2) *Y_1_* = +99.55 − 3.13X_1_ + 4.12X_2_ − 0.85X_3_ + 0.66X_4_ − 1.56X_5_ − 13.20X_1_^2^ − 16.19X_2_^2^ − 15.48X_3_^2^ − 15.46X_4_^2^ − 8.32X_5_^2^ − 1.42X_1_X_2_ − 0.37X_1_X_3_ − 1.89X_1_X_4_ − 3.17.44X_1_X_5_ − 2.06X_2_X_3_ − 1.86X_2_X_4_ + 1.84X_2_X_5_ + 2.17X_3_X_4_ + 4.47X_3_X_5_ − 2.63X_4_X_5,_

From Eq. (2), positive signs represented synergy among the independent variables while negative symbols represent antagonistic effects. therefore, X_2_, X_4_, X_2_X_5_, X_3_X_4_, and X_3_X_5_, represented synergistic effects while X_1_, X_3_, X_5_, X_1_^2^, X_2_^2^, X_3_^2^, X_4_^2^, X_5_^2^, X_1_X_2_, X_1_X_3_, X_1_X_4,_ X_1_X_5,_ X_2_X_3,_ X_2_X_4,_ and X_4_X_5_ were the antagonistic effects among the factors influencing phenanthrene degradation.

Based on the analysis of variance (ANOVA), coefficient of variation (R^2^) value of 0.9858 has shown good data correlation during phenanthrene degradation by *C. sakazakii* MM045. Meanwhile, the statistical model was found significant considering *F-*value of 100.86, whereas p *> F* value of <0.0001 only displayed 0.01% chance for large *F* value to occur due to the fitness of regression model to the experimental data. Hence, *Cronobacter sakazakii* MM045 was proven to be largely dependent on all five independent variables to have a better PAHs degradation response.

**Fig. 1:**
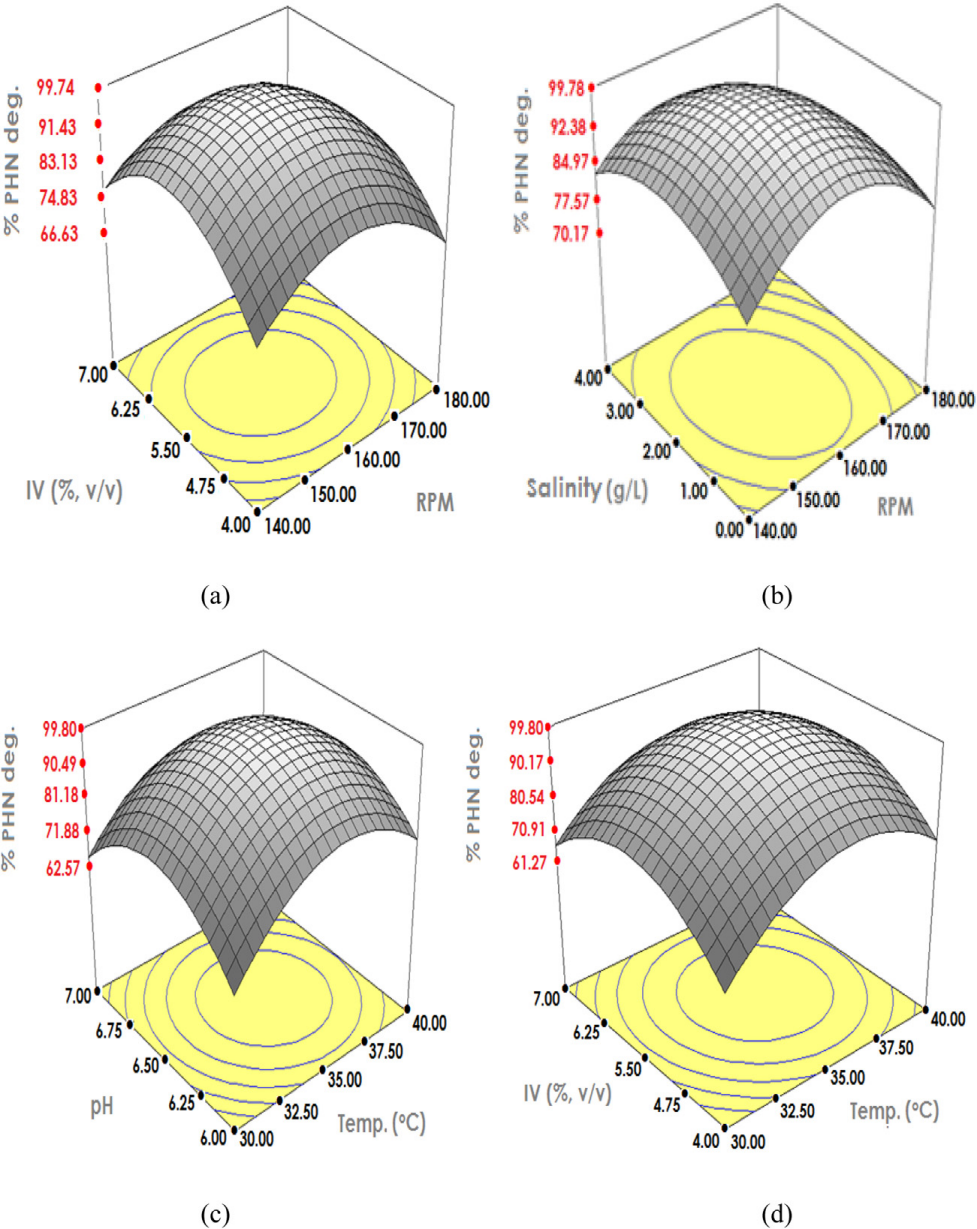

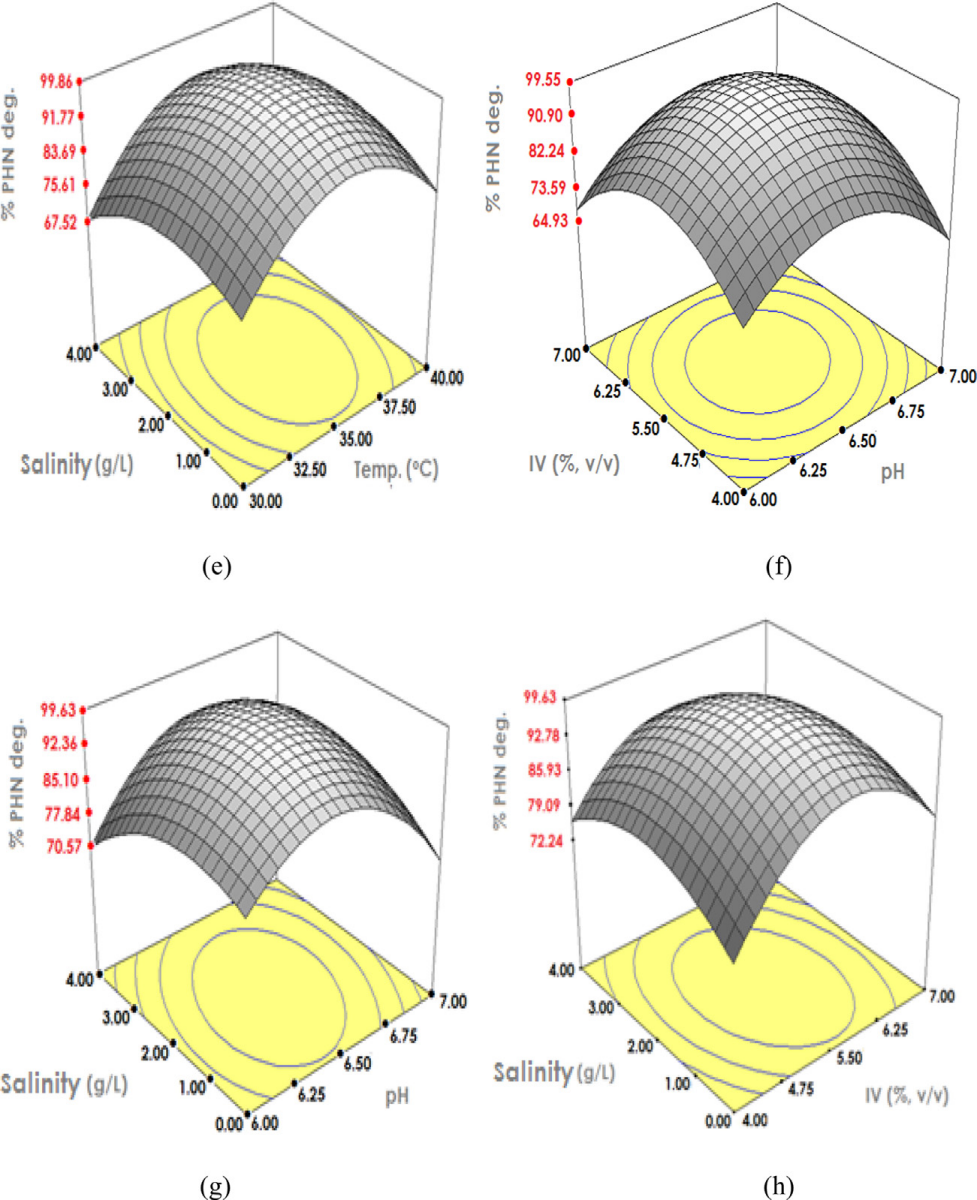
Interacting structures of phenanthrene degradation by *C. sakazakii* MM045 involving (a) IV & rpm, (b) Salinity & rpm, (c) pH & temperature, (d) IV & temperature, (e) Salinity & temperature, (f) IV & pH, (g) Salinity & pH, (h) Salinity & IV.

Considering the predicted compressibility response for phenanthrene degradation by *C. sakazakii* MM045, centre points of all interacting factors recorded significant response (Fig. 1). Nonetheless, such response decreased when the factors shifted toward the axial points with results validated by repeated experiments. Better prediction by the quadratic model was obtained based on the close gap between model prediction and actual degradation response. Consequently, complete phenanthrene degradation was attained at culture conditions of 158 rpm, 35 °C, pH 6.46, 5.50% v/v IV and 1.69 g/L NaCl.

### Results of pyrene optimisation

The same interpretation and experimental treatment for phenanthrene degradation was employed to pyrene using *C. sakazakii* MM045 (Table 1). Model quadratic equation for pyrene degradation was mathematically represented as:

(3) *Y_2_* = +99.26 − 2.21X_1_ − 6.75X_2_ − 3.49X_3_ − 2.95X_4_ − 5.41X_5_ − 13.72X_1_^2^ − 14.49X_2_^2^ − 12.37X_3_^2^ − 14.44X_4_^2^ − 10.96X_5_^2^ + 8.26X_1_X_2_ + 2.48X_1_X_3_ − 2.20X_1_X_4_ − 2.35X_1_X_5_ − 1.98X_2_X_3_ − 3.41X_2_X_4_ + 1.91X_2_X_5_ + 2.45X_3_X_4_ + 0.12X_3_X_5_ + 3.61X_4_X_5_

**Fig. 2:**
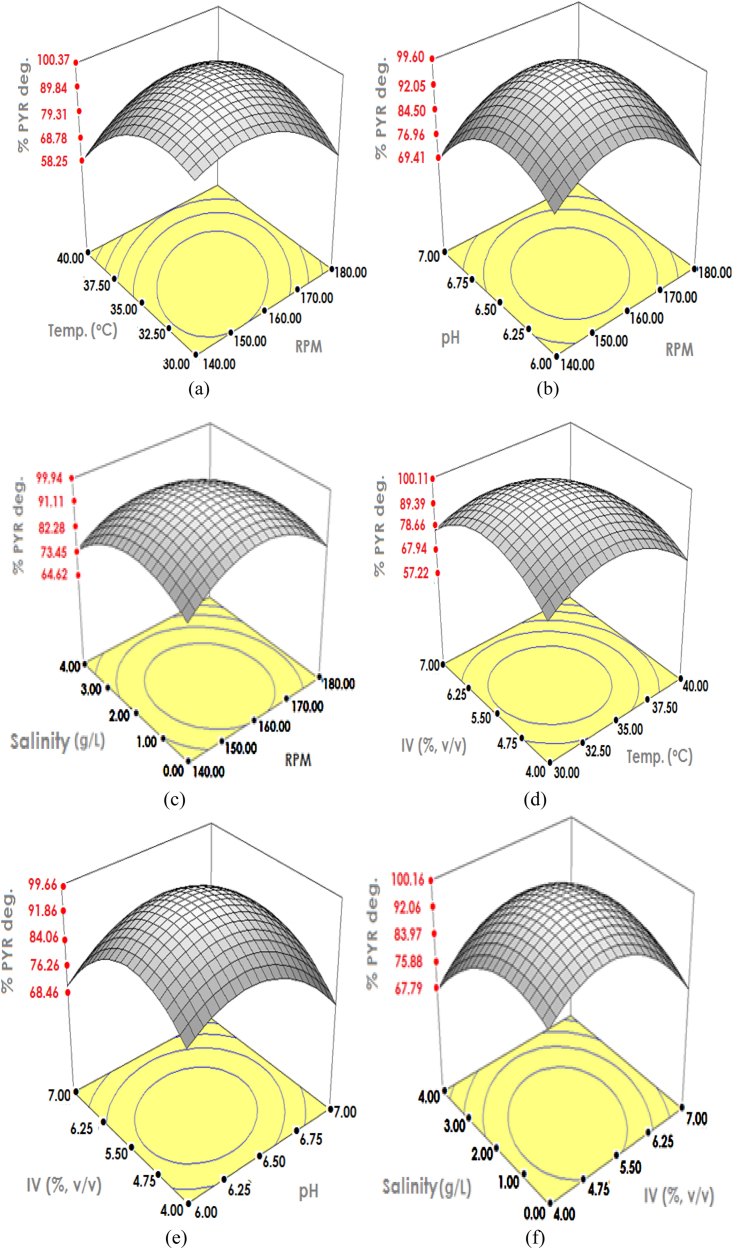
Three dimensional structures indicating pyrene degradation response of *C. sakazakii* MM045 based on interactions among (a) temp. & rpm, (b) pH & rpm, (c) Salinity & rpm, (d) IV & temp., (e) IV & pH, (f) Salinity & IV.

Correlation between the predicted *C. sakazakii* MM045 response in degrading pyrene and that of actual response was found highly significant (p < 0.0001) with the R^2^ value of 0.9728 and *F-*value of 51.85. The optimum pyrene degradation response by *C. sakazakii* MM045 was attained at 158 rpm, 35 °C, pH 6.46, 5.50% v/v IV, and 1.69 g/L NaCl (Fig. 2). Subsequently, temperature, IV, salinity, pH and agitation were observed to significantly enhance pyrene degradation response by *C. sakazakii* MM045 and final results were validated accordingly.

### Metabolites extraction, purification and identification (Steps 6–8)

After 12 h biodegradation period, phenanthrene and pyrene metabolites were separately extracted using liquid to liquid extraction process [46]. *Cronobacter sakazakii* cells were initially removed by centrifuging the culture for 15 min at 4000 rpm. Excess PAHs crystals were then removed by filtration with the filtrate adjusted to pH 2.0. The extraction was done using diethyl ether solvent and separating funnel where extracted moisture was dried with anhydrous sodium sulphate, which was then concentrated to 3 ml volume. Further extract purification process was conducted using column chromatography with silica gel as the stationary phase and *n*-hexane as the mobile phase [30]. Solvent from the pure extracts was dried by evaporation and then re-dissolved in 1 ml methanol prior to GC–MS which was proven as reliable identification technique for PAHs metabolites [16].

Gas Chromatography Mass Spectrophotometer (GC–MS) analyses for each phenanthrene and pyrene were carried out using thermoscientific TG-5MS column (30 m length, 0.25 mm i.d., 0.25 μm film). Helium carrier gas was injected at 1 ml/min using programmable split-less injector with an injection volume of 1 μl. The applied initial temperature of 50 °C was maintained for 3 min with the inlet temperature of 250 °C. Ramping at temperatures ranging from 10 °C to 320 °C/min was further done for 10 min. PAHs intermediate metabolites were identified by mass spectra (TQS Quantum XLS) using monitoring mode scan of 35–500 u and their comparisons to NIST database.

### Results of identified phenanthrene and pyrene degradation metabolites

**Fig. 3:**
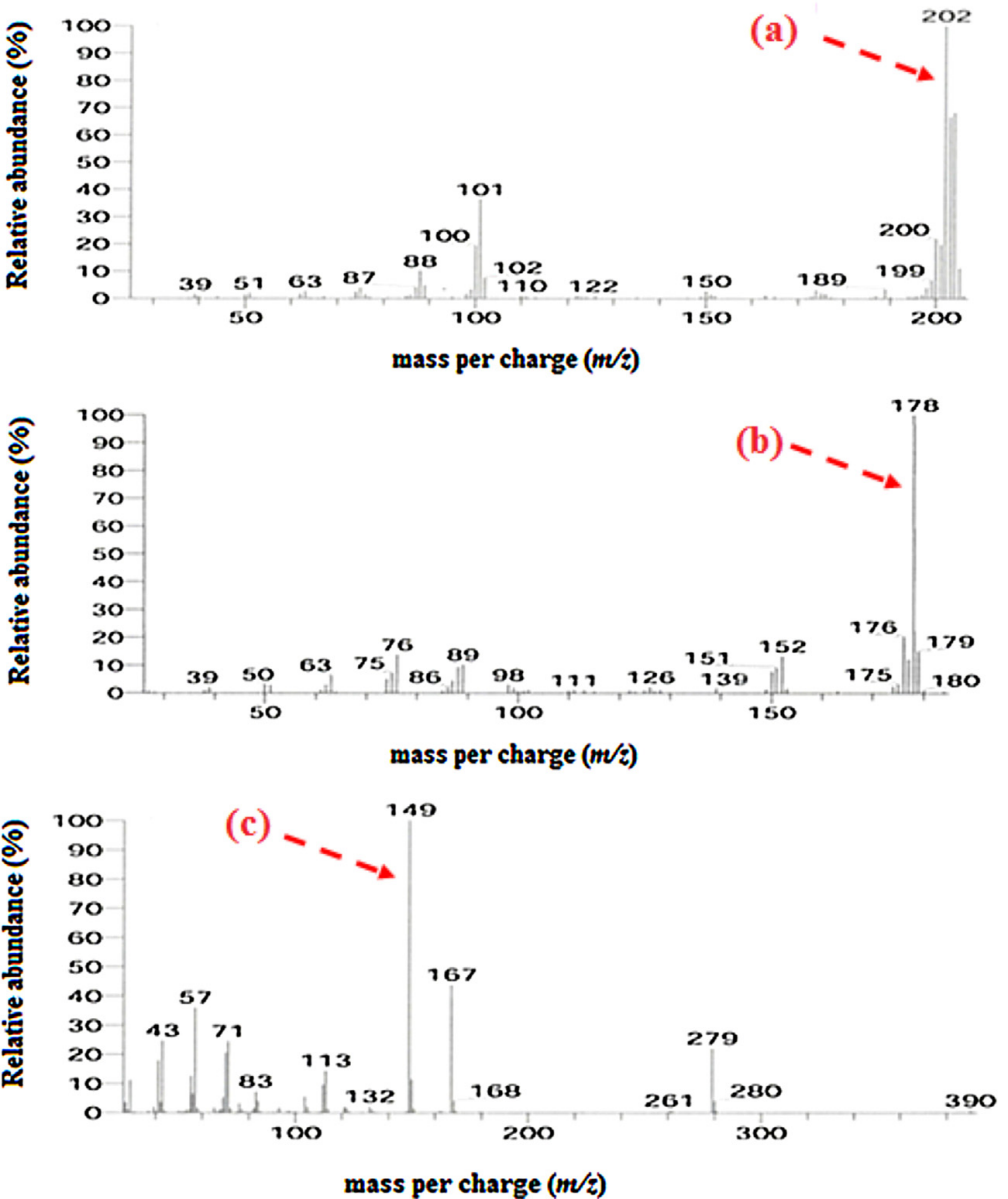

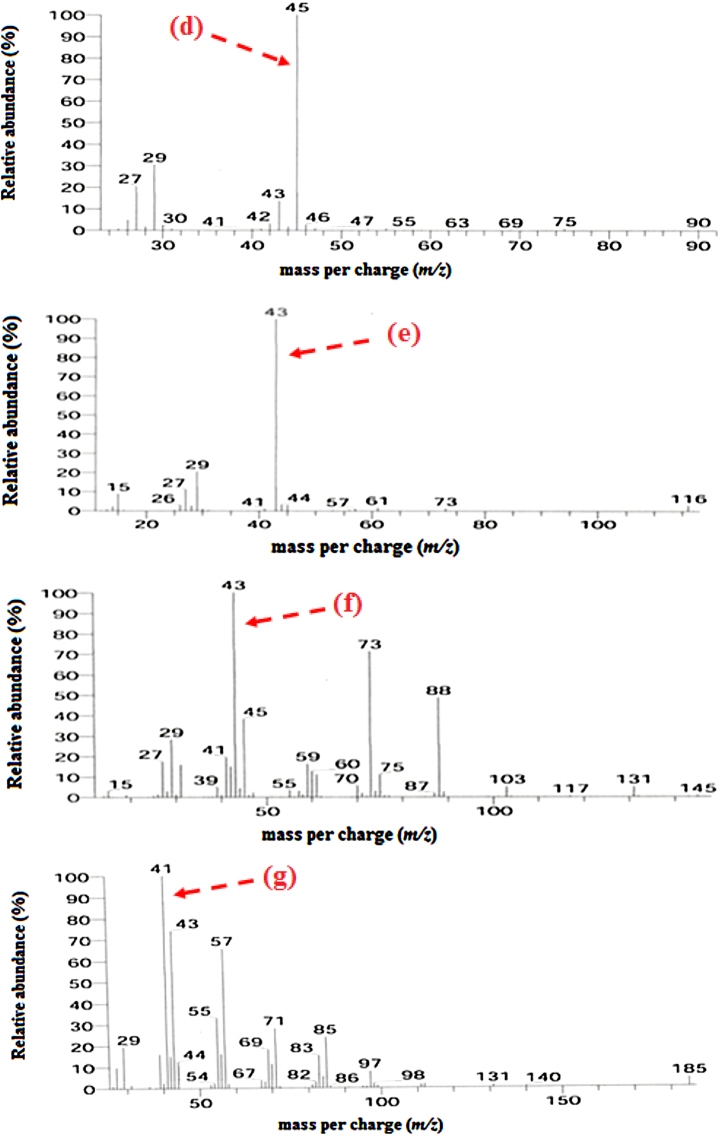
Mass spectra of intermediate metabolites identified from phenanthrene and pyrene degradation by *C. sakazakii* MM045 involving (a) pyrene *cis*-4,5-dihydrodiol, (b) 3,4-dihydroxy phenathrene, (c) pthalate, (d) lactic acid (e) pyruvic acid, (f) acetic acid, and (g) oxalic acid.

Degradation metabolites were identified by GC–MS after phenanthrene and pyrene were degraded by *C. sakazakii* MM045 (Fig. 3). In terms of phenanthrene metabolites, 3,4-dihydroxyphenathrene, phthalate, pyruvic acid, acetic acid and oxalic acid with GC abundance of 57%, 27%, 4%, 6%, and 3% were obtained at 19.13, 25.82, 4.26, 2.18 and 14.20 min, respectively. Meanwhile for pyrene degrading metabolites, pyrene *cis*-4,5-dihydrodiol, 3,4-dihydroxyphenanthrene, phthalate, pyruvic acid, acetic acid and lactic acid with the abundance of 8%, 56%, 19%, 15%, 10%, 13% GC were identified at 22.62, 19.12, 25.81, 3.47, 2.17 and 2.64 min, respectively (Fig. 3).

## Additional information

### Background of the study

Polycyclic aromatic hydrocarbons (PAHs) widely spread organic pollutants containing more than one benzene ring arranged in angular, linear or cluster positions [8]. PAHs form significant component of used engine oil through incomplete combustion process [42]. Therefore, the contamination of used engine oil within soil environment may negatively affect living ecosystems since PAHs are persistent, bio-accumulative, and toxic compounds [14]. The fate of PAHs within an environment is largely depends on their amount of benzene rings and the influence from environmental factors [8].

Many studies have highlighted the capabilities of several PAHs degrading bacteria and their biochemical pathways especially the mechanism of their ring oxidation and co-metabolism [22], [23]. There were also similar studies conducted that examined the capabilities of bacteria from used engine oil source in utilising PAHs as the sole carbon source [2], [39]. However, none of these studies investigated *Cronobacter sakazakii* despite the established evidence of their environmental distribution as most studies only highlighted their medical applications [24].

The environmental distribution of bacteria that include *C. sakazakii* helps in degrading highly dangerous organic pollutants such as PAHs [17]. However, this degradation may be negatively affected by environmental influences such as temperature, pH, salinity and microbial population [27]. The extremely low or high values of each influencing factor can minimise the efficiency of PAH degradation due to high microbial sensitivity [27]. In order to achieve better degradation outcome, these influencing factors have to be optimised to provide a more efficient PAH biodegradation result. Response surface methodology (RSM) based on central composite design (CCD) is an established mathematical approach that is able to achieve such optimisation by analysing the modelling effects of multiple variables [43]. Thus, this method has been successfully employed for the optimisation of PAHs microbial degradation [26].

Considering the environmental challenges in soil contamination especially PAHs toxicity to ecosystem and the difficulty of slow degradation faced by the reported microorganisms, this study intends to explore the potentiality of *C. sakazakii* MM045 as an effective alternative for PAHs biodegradation. Biodegradation ability can be enhanced using RSM (CCD), which will further aid the GC–MS to identify PAHs biodegradation metabolites. Phenanthrene and pyrene were selected to represents low and high molecular weight of PAHs.

### Discussion

Environmental accumulation of phenanthrene and pyrene seriously affects the living ecosystem [21]. In an earlier study conducted by Darma et al. [12], *C. sakazakii* MM045 has degraded 75.2% and 54.3% of phenanthrene and pyrene within 24 h despite limitations faced by culture conditions. This bacterium has the acclimatisation period of five and three hours for phenanthrene and pyrene, which might underestimate its degradation potential. This limitation therefore requires further optimisation of factors such as salinity, pH, temperature, agitation, and inoculums volume (IV) to enhance degradation response.

Regarding initial salinity effect, both phenanthrene and pyrene were rapidly degraded at moderate NaCl concentration of 1.69 g/L (Fig. 1, Fig. 2). Such salinity requirement was observed improving the *Cronobacter* buffering system, which in turn enhanced the acceleration of PAHs degradation [25]. However, high salinity concentration increased *C. sakazakii* lag time and caused eventual decrease in degradation rate due to salting out effect. Consequently, optimum PAHs degradation was achieved at appropriate salinity value of 1.69 g/L as concentration above 5 g/L reduces *Cronobacter* catabolic functions [38]. Additionally, the moderate salinity requirement by *C. sakazakii* MM045 might be due to the presence of several other inorganic salts in MS medium.

Among influencing factors, pH was selected due to its rare documentation on PAHs biodegradation (Pawar [32]). During CCD optimisation, both phenanthrene and pyrene were rapidly degraded at pH value of 6.46 (Fig. 1, Fig. 2). These values are close to neutral pH, which were mostly required by PAHs degrading bacteria [10]. However, changes in culture pH to lower acidic value have produced hydroxyl radicals that de-activated the PAHs degrading enzymes [1]. Moreover, increasing the pH to alkaline had caused eventual increase in PAHs toxicity, which inhibited *Cronobacter* growth [13]. Therefore, pH value of 6.46 was recorded to tremendously enhance the degradation process.

Another factor influencing PAHs biodegradation is temperature [7]. Temperature value of 35 °C was observed to significantly enhance complete phenanthrene and pyrene degradations from 75.2% and 54.3%, respectively (Fig. 1, Fig. 2). This has further strengthened *C. sakazakii* MM045 as mesophilic PAHs degrading bacterium that strives best in between 30 °C and 40 °C [44]. It was found that PAHs toxicity increased as the biodegradation temperature increases above 40 °C, whereas lowering the temperature below 30 °C has decreased the functions of degrading enzymes [4]. Moreover, the optimum temperature enhanced better enzymatic degradation function, which makes *C. sakazakii* MM045 a more effective PAHs degrader [38].

Agitation, being another contributing factor, improved rapid PAHs degradation by ensuring proper culture mix and adequate oxygenation [6], [7]. Agitation value of 158 rpm during CCD optimisation contributed towards achieving 100% phenanthrene and pyrene degradations. It was further observed that lowering the agitation value below 120 rpm caused inadequate oxygenation, which resulted in very low PAHs degradation (Fig. 1, Fig. 2). When such agitation is raised above 200 rpm, many *Cronobacter* cells died due to shear stress, reducing PAHs degradation tremendously. Therefore, 158 rpm is the best suitably agitation value required by *C. sakazakii* MM045 to rapidly degrade PAHs as their initial degradation step is oxidation process [35].

Inoculums volume (IV) is another limiting factor in the PAHs biodegradation [35]. The CCD optimisation showed 5.50% (v/v) of 10^6^*C. sakazakii* MM045 cells, which has also contributed to the complete phenanthrene and pyrene degradations (Fig. 1, Fig. 2). Despite the differences in phenanthrene and pyrene concentrations, optimum IV value remained the same, allowing both high and low molecular weight of PAHs to be successfully degraded. Decreasing IV below 4% has resulted in limited PAHs degradation due to structural toxicity effects of PAHs, whereas the increase in IV value above 7% v/v has caused the competition among *Cronobacter* cells. This has further resulted in significant population loss while the only surviving cells were inhibited by the PAHs toxicity.

**Fig. 4:**
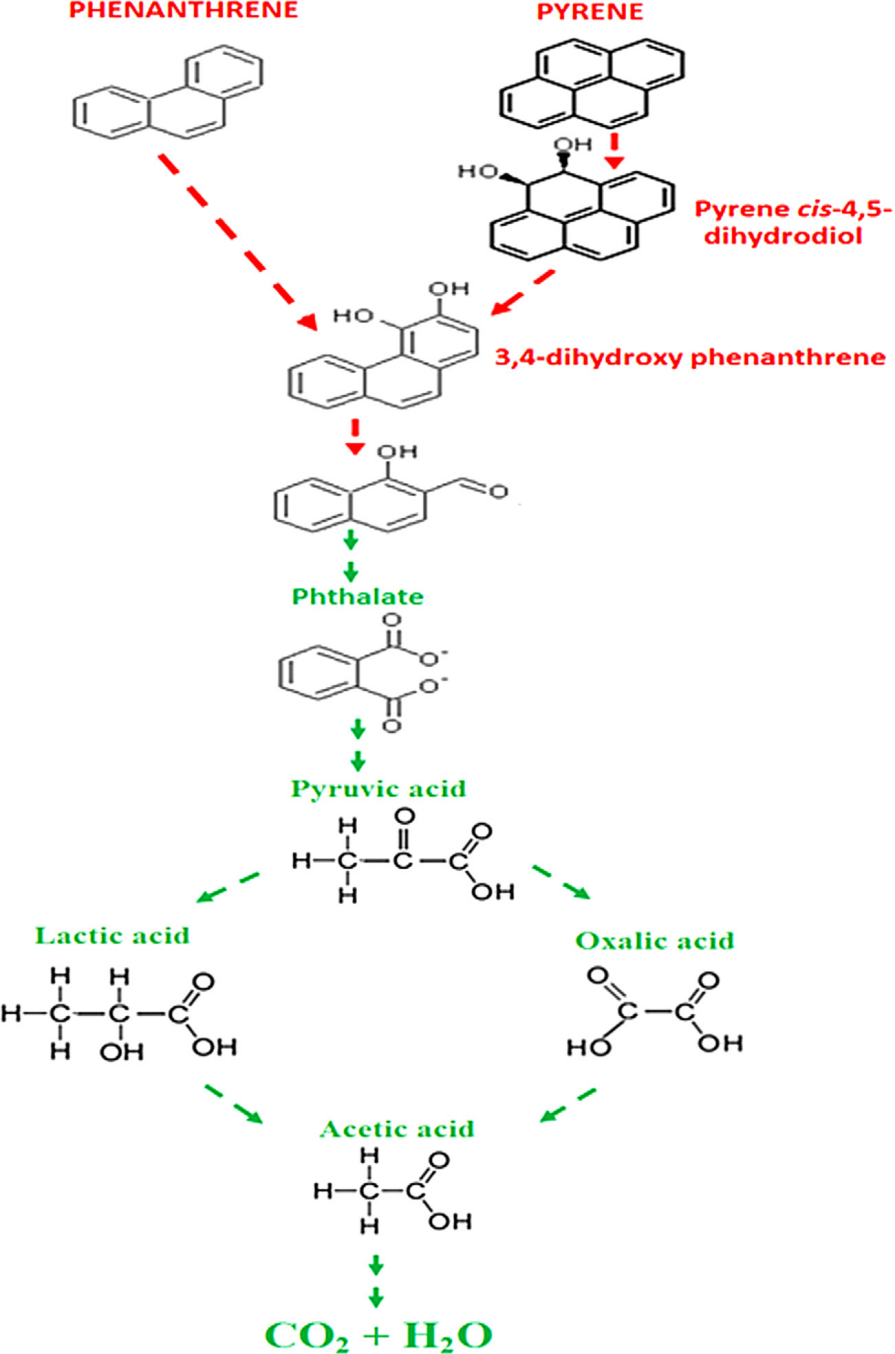
Proposed phenanthrene and pyrene degradation pathway using seven intermediate metabolites generated by *C. sakazakii* MM045 as; pyrene *cis*-4,5-dihydrodiol, 3,4-dihydroxy phenathrene, pthalate, pyruvic acid, oxalic acid, lactic acid, and acetic acid.

Regarding the intermediate biodegradation metabolites identified, pyrene *cis*-4,5-dihydrodiol was obtained as the initial pyrene metabolite that demonstrates *C. sakazakii* MM045 dioxygenate C_4_ and C_5_ of pyrene (Fig. 4). Moreover, phenanthrene was dioxygenated at C_3_ and C_4_, thereby producing 3,4-dihydroxyphenathrene as the initial metabolite (Fig. 4). These dioxygenations were followed by the dehydrogenation of initial products through a series of convergent pathways where phthalate metabolite was obtained [35]. Further catabolism of phthalic acid product was carried out through ring cleavage and dioxygenation where pyruvic acid was obtained, which later reduced to lactic acid, acetic acid and oxalic acid [28]. This confirms that both phenanthrene and pyrene were completely degraded by *C. sakazakii* MM045, which produced less hazardous metabolites of commercial importance [20], [31].

Considering the combined influence of degradation factors, environmental *C. sakazakii* might play a central role during commercial *in-situ* and *ex-situ* PAHs biodegradation. The study has shown that only 10^6^*C. sakazakii* cells are required to efficiently degrade PAHs at water content of 5.5%. Such water content is the most suitable for *in-situ* biodegradation as poor oxygenation has been reported at high water content [41]. Furthermore, optimum agitation value of 158 rpm has enhanced adequate oxygenation sustaining the maximum PAHs degradation. This can be proven during environment application through the provision of appropriate design of venting wells and blower equipment ensuring that the bacterium receives enough oxygenation [41]. Additionally, 35 °C might be the optimum temperature for *in-situ* and *ex-situ* applications as bioventing operates best at temperature ranging from 30 °C to 40 °C that is obtainable on soil environment [41].

Another degradation factor that enhances the potential of *C. sakazakii* MM045 is pH value of 6.46. This pH has fallen close to the most suitable value of 6.5 used for environmental PAHs biodegradation [3]. Moreover, pH adjustment can be made during both *in-situ* and *ex-situ* application by adding either lime or sulphur when PAHs radically alter the environmental pH [11]. Furthermore, Ulrich et al. [40] suggested that 1.69 g/L is among the best optimum salinity content required for environmental biodegradation. Hence, the contribution of all optimised factors might help in developing commercial remediation strategies that is cost effective and protect ecological habitats for indigenous organisms. This also provides several opportunities to develop and market seed culture for *C. sakazakii* MM045 as the previous report has estimated $180,000 as the cost of excavation, removal and backfilling PAHs contaminated soil with clean soil [5]. Additionally, *on site* burial of 1 m depth PAHs contaminated soil will cost about $100,000, whereas bioremediation alternative only costs $38,000 despite the slow degradation phase previously reported [5]. Therefore, environmental application of *C. sakazakii* MM045 provides attractive opportunities as the bacterium rapidly initiates PAHs degradation within three hours.

This study further strengthened that *C. sakazakii* MM045 is a potentially effective bacterium that completely degrades high and low molecular weight of PAHs within 24 h. This bacterium has a wide environmental distribution despite non existing report on its PAHs degradation capability [24]. Moreover, *C. sakazakii* MM045 has shown similar degradation pattern as those of *Pseudomonas, Burkholderia*, *Sphingomonas,* and *Mycobacterium* species [35]. Furthermore, *C. sakazakii* MM045 as a single strain was observed to be more efficient than many PAHs degrading consortia previously reported [35]. In a previous report, only 100 mg/L of phenanthrene has been mostly degraded rapidly (97.5%) by *Sphingomonas* sp. within 24 h in optimised culture medium [37]. Furthermore, 48% pyrene was reported to be rapidly degraded by *Mycobacterium* sp. after 72 h of incubation even with higher population of 1.5 × 10^6^ cells/ml [17]. This has further highlighted the good metabolic strength of *C. sakazakii* MM045 in degrading PAHs, which might be previously endorsed from the contaminated environment [24].

### Conclusion

*Cronobacter sakazakii* MM045 has shown to rapidly and effectively degrade phenanthrene and pyrene as the environmental pollutants. CCD optimisation also significantly improved the strength of *C. sakazakii* MM045 to achieve complete degradation. The pathways followed by *C. sakazakii* MM045 in degrading phenanthrene and pyrene were confirmed through the identification of relevant metabolites. Therefore, it can be concluded that *Cronobacter sakazakii* MM045 is a potential biodegrading candidate that can rapidly detoxify PAHs environmental contamination.

## Figures and Tables

**Table 1:** Experimental set-up for phenanthrene and pyrene degradation by *C. sakazakii* MM045 based on RSM (CCD) involving five independent variables.

Std No.	Factors					Phenanthrene Degradation	Pyrene Degradtion
	RPM	Temp.	pH	IV	NaCl		
1	140.0	30.0	6.0	4.0	0.0	30.2	57.6
2	180.0	30.0	6.0	4.0	0.0	32.2	52.4
3	140.0	40.0	6.0	4.0	0.0	50.0	35.6
4	180.0	40.0	6.0	4.0	0.0	40.0	60.6
5	140.0	30.0	7.0	4.0	0.0	15.2	48.8
6	180.0	30.0	7.0	4.0	0.0	26.0	50.8
7	140.0	40.0	7.0	4.0	0.0	20.2	23.8
8	180.0	40.0	7.0	4.0	0.0	26.4	42.0
9	140.0	30.0	6.0	7.0	0.0	34.8	57.2
10	180.0	30.0	6.0	7.0	0.0	35.4	36.6
11	140.0	40.0	6.0	7.0	0.0	49.2	24.6
12	180.0	40.0	6.0	7.0	0.0	37.4	16.1
13	140.0	30.0	7.0	7.0	0.0	34.4	51.8
14	180.0	30.0	7.0	7.0	0.0	33.8	35.6
15	140.0	40.0	7.0	7.0	0.0	33.2	14.8
16	180.0	40.0	7.0	7.0	0.0	33.4	28.4
17	140.0	30.0	6.0	4.0	4.0	19.4	52.0
18	180.0	30.0	6.0	4.0	4.0	20.2	15.2
19	140.0	40.0	6.0	4.0	4.0	43.8	32.8
20	180.0	40.0	6.0	4.0	4.0	40.2	36.3
21	140.0	30.0	7.0	4.0	4.0	33.8	39.6
22	180.0	30.0	7.0	4.0	4.0	20.6	15.0
23	140.0	40.0	7.0	4.0	4.0	50.0	8.4
24	180.0	40.0	7.0	4.0	4.0	34.0	32.2
25	140.0	30.0	6.0	7.0	4.0	24.2	52.0
26	180.0	30.0	6.0	7.0	4.0	11.8	16.8
27	140.0	40.0	6.0	7.0	4.0	35.4	21.2
28	180.0	40.0	6.0	7.0	4.0	22.4	30.0
29	140.0	30.0	7.0	7.0	4.0	40.4	56.0
30	180.0	30.0	7.0	7.0	4.0	21.8	31.2
31	140.0	40.0	7.0	7.0	4.0	50.6	7.4
32	180.0	40.0	7.0	7.0	4.0	22.6	25.8
33	112.4	35.0	6.5	5.5	2.0	28.0	25.6
34	207.6	35.0	6.5	5.5	2.0	15.8	10.0
35	160.0	23.1	6.5	5.5	2.0	0.0	26.8
36	160.0	46.9	6.5	5.5	2.0	10.0	0.0
37	160.0	35.0	5.3	5.5	2.0	10.4	39.2
38	160.0	35.0	7.7	5.5	2.0	7.6	11.6
39	160.0	35.0	6.5	1.9	2.0	7.0	20.0
40	160.0	35.0	6.5	9.1	2.0	11.2	7.4
41	160.0	35.0	6.5	5.5	−2.8	55.2	48.0
42	160.0	35.0	6.5	5.5	6.8	43.8	18.8
43	160.0	35.0	6.5	5.5	2.0	100.0	89.0
44	160.0	35.0	6.5	5.5	2.0	92.5	100.0
45	160.0	35.0	6.5	5.5	2.0	100.0	100.0
46	160.0	35.0	6.5	5.5	2.0	100.0	100.0
47	160.0	35.0	6.5	5.5	2.0	100.0	100.0
48	160.0	35.0	6.5	5.5	2.0	100.0	100.0
49	160.0	35.0	6.5	5.5	2.0	100.0	100.0
50	160.0	35.0	6.5	5.5	2.0	100.0	100.0
